# Decoupling and Parameter Extraction Methods for Conical Micro-Motion Object Based on FMCW Lidar

**DOI:** 10.3390/s24061832

**Published:** 2024-03-13

**Authors:** Zhen Yang, Yufan Yang, Manguo Liu, Yuan Wei, Yong Zhang, Jianlong Zhang, Xue Liu, Xin Dai

**Affiliations:** 1 Department of Optoelectronic Information Science and Technology, Harbin Institute of Technology, Harbin 150080, China; sailoryz@hit.edu.cn (Z.Y.); 23s021018@stu.hit.edu.cn (Y.Y.); liuxue032243@163.com (X.L.); daixin4236@163.com (X.D.); 2 Xi’an Modern Control Technology Research Institute, Xi’an 710065, China; 18192226519@163.com; 3 Beijing Aerospace Automatic Control Institute, Beijing 100854, China; wei-0330@163.com

**Keywords:** micro-motion, decoupling, FMCW lidar, laser micro-Doppler, range profile

## Abstract

Micro-Doppler time–frequency analysis has been regarded as an important parameter extraction method for conical micro-motion objects. However, the micro-Doppler effect caused by micro-motion can modulate the frequency of lidar echo, leading to coupling between structure and micro-motion parameters. Therefore, it is difficult to extract parameters for micro-motion cones. We propose a new method for parameter extraction by combining the range profile of a micro-motion cone and the micro-Doppler time–frequency spectrum. This method can effectively decouple and accurately extract the structure and the micro-motion parameters of cones. Compared with traditional time–frequency analysis methods, the accuracy of parameter extraction is higher, and the information is richer. Firstly, the range profile of the micro-motion cone was obtained by using an FMCW (Frequency Modulated Continuous Wave) lidar based on simulation. Secondly, quantitative analysis was conducted on the edge features of the range profile and the micro-Doppler time–frequency spectrum. Finally, the parameters of the micro-motion cone were extracted based on the proposed decoupling parameter extraction method. The results show that our method can effectively extract the cone height, the base radius, the precession angle, the spin frequency, and the gravity center height within the range of a lidar LOS (line of sight) angle from 20° to 65°. The average absolute percentage error can reach below 10%. The method proposed in this paper not only enriches the detection information regarding micro-motion cones, but also improves the accuracy of parameter extraction and establishes a foundation for classification and recognition. It provides a new technical approach for laser micro-Doppler detection in accurate recognition.

## 1. Introduction

At present, high-resolution lidar measurement technology plays an important role in fields such as ocean exploration [1] and atmospheric environment monitoring [2]. Laser micro-Doppler technology, as one of the high-resolution measurement methods, has been widely used in various fields such as vital sign detection [3,4,5,6], drone detection [7,8], automotive radar detection [9], vibration measurement [10], and bird recognition [11]. When a radar emits electromagnetic waves onto the surface of a moving object, the echo frequency will undergo a Doppler shift compared to the emitting signal. In addition to the movement of the main body, the micro-motion of some components can cause additional frequency modulation to the echo and generate side frequencies near the original Doppler frequency shift, which is known as the micro-Doppler effect [12]. The cone object undergoes regular micro-motion in addition to translational flight, which has attracted much attention.

Conical micro-motion information can be measured by using the micro-Doppler time–frequency spectrum [13]. The peak components in the spectrum represent the micro-Doppler frequency shift caused by the radial velocity of the micro-motion object. The width of the frequency shift provides an estimate of the dispersion caused by the micro-Doppler effect. The difference in curve smoothness can be used to separate the micro-Doppler curves of the cone scattering points. The empirical mode decomposition method can be used to detect and estimate the precession frequency [14]. For cones with occlusion effects, Zhou et al. [15] proposed a new algorithm of coherent single-range Doppler interferometry (CSRDI)-modified general parameterized time–frequency (GPTF) to extract micro-Doppler curves and estimate parameters. Qin et al. [16] also extracted micro-Doppler characteristic parameters of rotor aircraft blades through time–frequency analysis. Generally, the echo is subjected to short-time Fourier transform (STFT) to obtain the time–frequency spectrum. Hough transform is applied to extract the angle Doppler parameters from the time–frequency spectrum [17]. In addition, matching pursuit (MP) has also been proven to be an effective signal decomposition method [18], which decomposes any signal into a combination of basic waveforms. So, it is suitable for reconstructing different types of micro-Doppler signals and extracting micro-motion parameters. Liang et al. [19] also proposed a method for extracting vibration frequency and amplitude parameters based on the slow time envelope features of bottom vibration objects.

However, the extracted parameter information obtained through time–frequency transformation is not rich enough. Therefore, it is necessary to obtain the one-dimensional range profile to extract structural parameters. The processing of range profiles on the obtained distance sequences is an important technical means for lidar detection and recognition. One-dimensional range profiles are important features of optical radar recognition, which can effectively represent the distribution of scattering centers along the lidar line of sight direction. They also obtain structure and shape information about the detected object [20]. Compared to the pulse lidar, the advantage of the FMCW lidar in distance measurement is that the frequency modulation laser source can be integrated into a chip [21], and the received beat frequency represents the distance and Doppler information about the object. Useful information about the object can be detected and extracted in this beat frequency domain [22]. Furthermore, the FMCW lidar does not need to consider the issue of the large surge currents generated in the pulse lidar [23]. The FMCW lidar based on coherent detection also has significant advantages in background noise and atmospheric attenuation [24]. By performing fast Fourier transform on the echo in the fast time domain, a slow time range profile can be obtained [25]. Detecting the micro-motion of objects in the laser band is more accurate compared to other bands, and the FMCW lidar can also be used to extract micro-Doppler feature information. Lee et al. [26] proposed a method for extracting high-frequency micro-Doppler features of the FMCW lidar based on the micro-motion object signal model. Peter et al. [27] proposed a new method that combined Cross Wigner Ville and Cross Wigner Hough transforms for the detection and parameter extraction of FMCW signals. However, the modulation of micro-Doppler echo frequency can also lead to offset and broadening effects of the range profile in the fast time domain. The coupling of range profiles between the micro-motion parameters and the structural parameters greatly affects the accuracy of parameter extraction and cone classification recognition. Therefore, research on how to decouple and extract accurate structural parameters from the micro-motion cone range profile is of great application significance.

To solve the difficulty of decoupling the conical motion range profile, this paper firstly obtained the range profile of conical micro-motion objects by an FMCW lidar based on simulation, quantitatively analyzed the micro-motion and echo models of conical objects, and then proposed a method for extracting the structure and micro-motion parameters of conical objects based on the micro-Doppler time–frequency spectrum and range profile. Finally, the decoupling and extraction of the structure and micro-motion parameters of conical objects were achieved.

## 2. Analysis of One-Dimensional Range Profile of Cone Based on FMCW Lidar

### 2.1. Dynamic Model Analysis of Precession Cone

**Figure 1:**
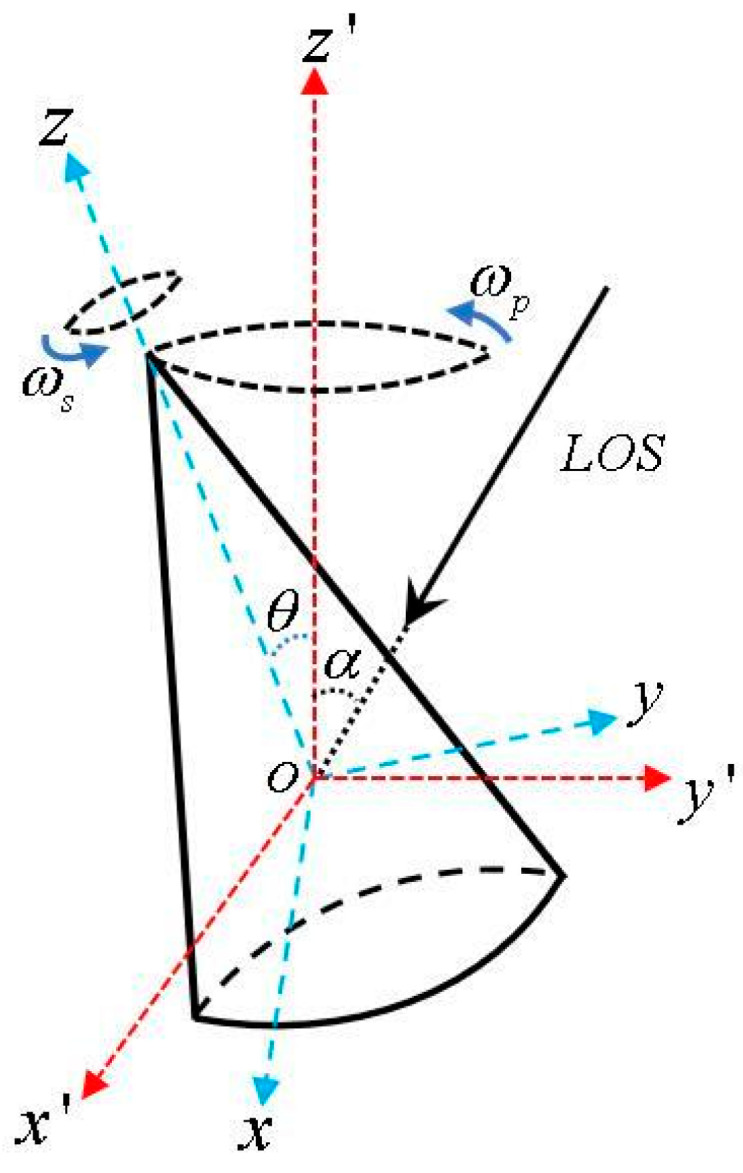
Mathematical model of conical micro-motion.

The schematic diagram of cone precession is shown in Figure 1. Point *o* is the center of mass, $\left(o,x^{\prime },y^{\prime },z^{\prime }\right)$ is the reference coordinate system, and (o,x,y,z) is the ontology coordinate system. *α* is the lidar line of sight angle. To simplify the operation, let *α* be in the *o y z* plane. The LOS angle unit vector is denoted as follows:

(1) $$\vec{n}=\left(0,\sin\alpha ,\cos\alpha \right)$$

When the cone performs a spin motion with an angular velocity of $\omega_{s}$ around its symmetry axis *oz*, it also performs a conical rotation motion with an angular velocity of $\omega_{p}$ around the axis *oz*′ in space. The angle between the conical rotation axis *oz*′ and the conical spin axis *oz* is the precession angle θ.

Assuming that the initial coordinates of a certain scattering point p on the cone in the reference frame is ${\vec{p}}_{0}=\left(x_{p},y_{p},z_{p}\right)$, then the coordinates of the scattering point p at the time t can be expressed as follows:

(2) $$\vec{p}\left(t\right)=R_{c}\left(t\right)R_{s}\left(t\right){\vec{p}}_{0}$$

where $\vec{p}\left(t\right)$ is the coordinate vector of scattering point p, $R_{s}\left(t\right)$ is the spin matrix, and $R_{c}\left(t\right)$ is the conical rotation matrix.

(3) $$R_{s}\left(t\right)=I+Q_{s}\sin\omega_{s}t+{Q_{s}}^{2}(1-\cos\omega_{s}t)$$

(4) $$R_{c}\left(t\right)=I+Q_{c}\sin\omega_{p}t+{Q_{c}}^{2}(1-\cos\omega_{p}t)$$

where **I** is the identity matrix, $Q_{s}$ is the oblique symmetry matrix corresponding to the spin axis, and $Q_{c}$ is the oblique symmetry matrix corresponding to the conical rotation axis.

(5) $$Q_{s}=\left[\begin{array}{ccc} 0 & -1 & 0\\ 1 & 0 & 0\\ 0 & 0 & 0 \end{array}\right]$$

(6) $$Q_{c}=\left[\begin{array}{ccc} 0 & -\cos\theta & \sin\theta \\ \cos\theta & 0 & 0\\ -\sin\theta & 0 & 0 \end{array}\right]$$

Substituting Equations (5) and (6) into Equations (3) and (4), respectively, then one obtains the following:

(7) $$R_{s}\left(t\right)=\left[\begin{array}{ccc} \cos(\omega_{s}t) & -\sin(\omega_{s}t) & 0\\ \sin(\omega_{s}t) & \cos(\omega_{s}t) & 0\\ 0 & 0 & 1 \end{array}\right]$$

(8) $$R_{c}\left(t\right)=\left[\begin{array}{ccc} \cos\omega_{p}t & -\cos\theta \sin\omega_{p}t & \sin\theta \sin\omega_{p}t\\ \cos\theta \sin\omega_{p}t & \cos^{2}\theta \left(\cos\omega_{p}t-1\right)+1 & \sin\theta \cos\theta \left(1-\cos\omega_{p}t\right)\\ -\sin\theta \sin\omega_{p}t & \sin\theta \cos\theta \left(\cos\omega_{p}t-1\right) & \sin^{2}\theta \left(\cos\omega_{p}t-1\right)+1 \end{array}\right]$$

The expression for the projection distance of any scattering point p on the LOS on the precession cone is obtained as follows:

(9) $$\begin{array}{c} r_{p}\left(t\right)=r_{0}+{\vec{n}}^{T}\vec{p}\left(t\right)\\ =r_{0}+\sin\alpha \left[\begin{array}{l} x_{p}\left(\cos\omega_{p}t\sin\omega_{s}t+\cos\theta \sin\omega_{p}t\sin\omega_{s}t\right)\\ y_{p}\left(+\cos^{2}\theta \left(\cos\omega_{p}t-1\right)\cos\omega_{s}t-\cos\theta \sin\omega_{p}t\sin\omega_{s}t+\cos\omega_{s}t\right)\\ +z_{p}\left(\sin\theta \sin\omega_{p}t\sin\omega_{s}t+\sin\theta \cos\theta \left(1-\cos\omega_{p}t\right)\cos\omega_{s}t\right) \end{array}\right]\\ +\cos\alpha \left[-x_{p}\sin\theta \sin\omega_{p}t+y_{p}\sin\theta \cos\theta \left(\cos\omega_{p}t-1\right)+z_{p}\left(\sin^{2}\theta \left(\cos\omega_{p}t-1\right)+1\right)\right] \end{array}$$

where $r_{p}\left(t\right)$ is scattering point p to projection distance, $r_{0}$ is scattering point p to radar initial projection distance, and ${\vec{n}}^{T}\vec{p}\left(t\right)$ is scattering point p to micro-motion projection distance variation.

### 2.2. FMCW Lidar for One-Dimensional Range Profile of Cone

**Figure 2:**
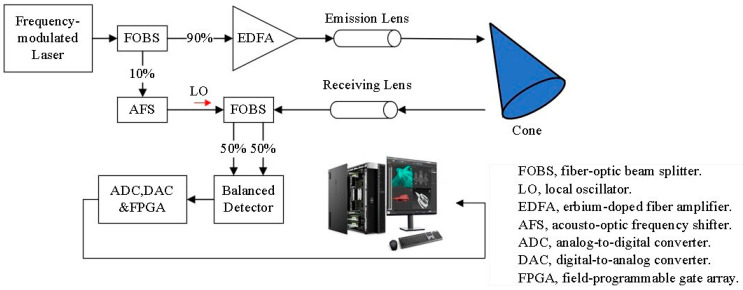
Schematic diagram of FMCW laser heterodyne interference ranging.

FMCW laser ranging is an absolute measurement technique based on self-heterodyne interferometry [28]. The basic ranging system is shown in Figure 2. The laser source is a frequency-modulated continuous beam, and the output laser is divided into two beams in a ratio of 1:9 by a beam splitter, in which 10% of the beam energy is used as the reference light and passes through an acousto-optic modulator, while 90% of the beam energy is used as the emission beam and passes through an amplifier. The emitted beam is incident from the emitting lens onto the surface of the measured object. The scattered echoes interfere with the reference beam in the beam combiner through the receiving lens, and the mixed beam is input 1:1 into the balanced detector to obtain the beat frequency signal. Finally, the signal is input into the computer through a signal acquisition card to display the time-domain and frequency-domain information about the beat frequency.

Assuming that the carrier frequency of the FMCW lidar transmission signal is $f_{0}$, the frequency modulation slope is *k*, the total time is *t*, the slow time is $t_{m}$, and the fast time is $\hat{t}$, then $t=\hat{t}+t_{m}$. The transmission signal *S* and the echo signal $S_{p}$ of any scattering point p on the cone are as follows:

(10) $$S\left(\hat{t},t_{m}\right)=A\exp\left[j2\pi (f_{0}t+\frac{1}{2}k{\hat{t}}^{2})\right]$$

(11) $$S_{p}\left(\hat{t},t_{m}\right)=\sigma_{p}A\exp\left\{j2\pi \left[f_{0}(t-t_{p})+\frac{1}{2}k{\left(\hat{t}-t_{p}\right)}^{2}\right]\right\}$$

where $t_{p}$ is the round-trip time from the lidar to point p; thus, $t_{p}=2r_{p}\left(t\right)/c$. The $r_{p}\left(t\right)$ is the projection distance of the scattering point p on the LOS obtained from Equation (9), and $\sigma_{p}$ is the scattering coefficient. *A* is amplitude.

The round-trip time between the cone and the lidar is $t_{ref}=2R_{\mathrm{ref}}/c$, roughly measured by the narrow bandwidth signal emitted by the laser source. The signal of the reference optical path can be expressed as follows:

(12) $$S_{ref}\left(\hat{t},t_{m}\right)=A\exp\left\{j2\pi \left[f_{0}(t-t_{ref})+\frac{1}{2}k{\left(\hat{t}-t_{ref}\right)}^{2}\right]\right\}$$

where $S_{ref}$ is the local oscillator reference beam.

Then, the echo and reference beam are mixed for interference, and the output beat frequency signal can be described as follows:

(13) $$S_{\mathrm{if}}\left(\hat{t},t_{m}\right)=\sigma_{p}\;A\exp\left\{j2\pi \left[\begin{array}{l} f_{0}(t_{p}-t_{ref})\\ +k(t_{p}-t_{ref})(t-t_{ref})\\ -\frac{1}{2}k{\left(t_{p}-t_{ref}\right)}^{2} \end{array}\right]\right\}$$

where $S_{\mathrm{if}}$ is the beat frequency beam.

Let $\Delta R=r_{p}(t)-R_{ref}$ and the pulse width of the transmission signal be $T_{p}$. Then, Fourier transform is performed on Equation (13) to obtain the frequency domain expression, which can be expressed as follows:

(14) $$\begin{array}{c} S_{f}\left(f,t_{m}\right)=T_{p}\sin c\left[T_{p}\left(f+2\frac{k}{c}\Delta R\right)\right]\\ \exp\left(-j\frac{4\pi k{\left(\Delta R\right)}^{2}}{c^{2}}\right)\exp\left(j\frac{4\pi f_{0}\Delta R}{c}\right)\exp\left(j\frac{4\pi kf\Delta R}{c}\right) \end{array}$$

The ΔR characterizes the distance variation in micro-motion scattering points relative to the position of the object body. The first phase in Equation (14) is the residual video phase (RVP), which is not beneficial for distance imaging. The second phase is the Doppler phase, which includes micro-Doppler information about scattering points. The third phase is the distance phase, which contains information on the distance changes for different scattering points relative to the position of the object body.

Considering the frequency domain expression as a sinc function with a peak position of f=−2kfΔR/c, the distance variation for each scattering point detected on the object corresponds to the peak position of the beat signal spectrum. Then, converting the frequency domain into distance by the equation of f=−2kfΔR/c, the one-dimensional range function of the precession cone is obtained.

### 2.3. Simulation Analysis of One-Dimensional Range Profile

**Figure 3:**
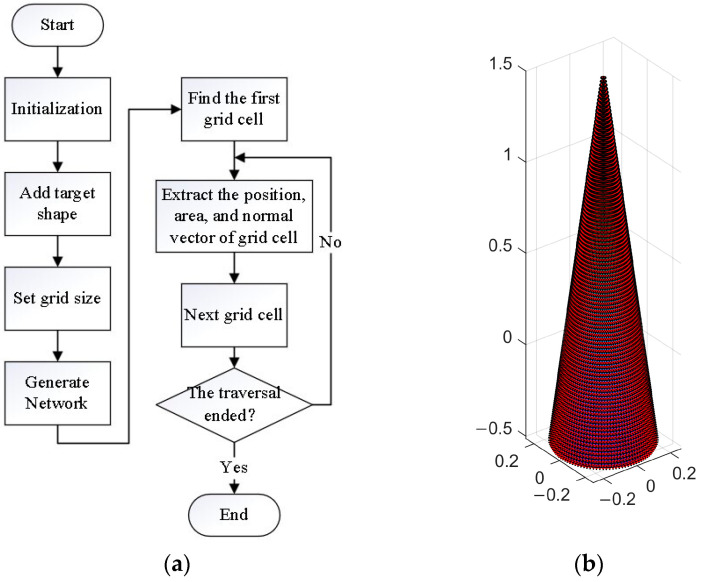
Flowchart and cone model. (**a**) Network model establishment flowchart; (**b**) cone 3D face element model.

The flowchart for establishing the network model is shown in Figure 3a. Firstly, the 3D mesh generation function is initialized and used to generate the 3D mesh of the cone. Then, read the target shape parameters are read and the generation function is used to transform a solid object into a three-dimensional surface. Finally, by reading the laser wavelength, the size of the object segmentation into small units is determined to obtain the surface network. The established three-dimensional network model of the cone is shown in Figure 3b, with a cone height of 2 m, a center of gravity height of 0.5 m, and a bottom radius of 0.25 m.

After finding a unit grid on the cone, the position, area, and normal direction of the unit are calculated, and then, iteration is performed through the entire cone to obtain the grid units of all areas. The network units are taken as scattering points on the conical surface for subsequent simulation.

**Figure 4:**
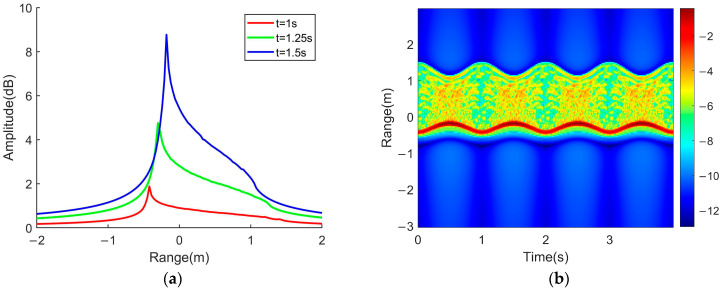
Range profile and range–time spectrum. (**a**) Cone’s one-dimensional range profile; (**b**) cone precession range-time spectrum.

The cone precession parameters and FMCW lidar emission parameters are shown in Table 1. Using Equation (14), the one-dimensional distance profiles of the precession cone at different times are simulated, and the distance–time map obtained by arranging the one-dimensional distance profiles at different times within the total time is shown in Figure 4.

Due to the periodic changes in peak intensity and the position of the one-dimensional range profile during the cone precession process, the edge width of the range profile, including the radial width information about the cone in the LOS, also undergoes changes periodically. Therefore, it is easy to extract precession period information through the changes in the range–time spectrum in the slow time domain.

## 3. Analysis of Parameter Decoupling and Extraction Based on Micro-Doppler Time–Frequency Spectrum and Range Profile

### 3.1. Acquisition and Simulation of Micro-Doppler Frequency Spectrum for the Micro-Motion Cone

Laser micro-Doppler detection also uses a heterodyne detection system of laser speed and distance measurement [29]. Using the laser heterodyne interference system in Figure 2, the laser source is replaced with a single-frequency laser of 1064 nm. The expression of the reference beam is changed from Equation (12) to the following:

(15) $$S\left(t\right)=A\exp(j2\pi f_{0}(t-t_{ref}))$$

The echo expression is described as follows:

(16) $$S_{p}\left(t\right)=A\exp(j2\pi f_{0}(t-t_{p}))$$

where $S_{p}$ is the echo single-frequency beam.

The expression of the intermediate frequency signal after mixing interference is as follows:

(17) $$S_{decp}\left(t\right)=\sigma_{p}\;A\exp(j2\pi \left(f_{0}(t_{p}-t_{ref})\right))$$

where $S_{decp}$ is the mixed signal beam.

The expression for the micro-Doppler spectrum obtained by performing Fourier transform on Equation (17) is as follows:

(18) $$S_{f}\left(f\right)=T_{p}\sin c(T_{p}f)\exp\left(j\frac{4\pi f_{0}\Delta R}{c}\right)$$

where $S_{f}$ is the frequency domain expression for mixed frequency signals.

**Figure 5:**
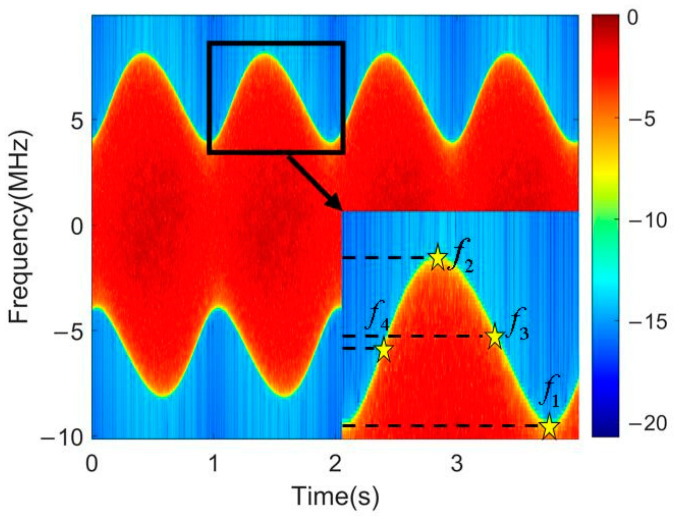
Simulation result of conical micro-Doppler time spectrum.

As can be seen from Equation (18), the phase term of the micro-Doppler spectrum expression is consistent with the Doppler phase term in Equation (14). According to Equation (18), the simulated cone micro-Doppler time spectrum is shown in Figure 5. The lidar and conical micro-motion parameters are the same as in Table 1.

The most obvious information in Figure 5 is the upper and lower edge curves, which represent the variation in the maximum micro-Doppler frequency shift with time at each moment. The maximum radial velocity of the conical micro-motion in the direction of LOS at each moment is located at the bottom edge of the cone. In other words, the maximum micro-Doppler frequency shift comes from the echo of the scattering points at the bottom edge. According to the dynamic model of the micro-motion cone, the expression for the temporal variation in micro-Doppler frequency at the scattering points on the bottom edge of the cone can be derived, which is described as follows:

(19) $$f_{D}\left(t;\phi \right)=\frac{2f_{0}}{c}\times \left\{\begin{array}{c} \rho \left(\omega +W\cos\theta \right)\sin\alpha \cos\omega t\cos\left(Wt+\phi \right)\\ -\rho W\cos\alpha \cos\left(Wt+\phi \right)\sin\theta \\ +\sin\alpha \sin\omega t\left[d\omega \sin\theta -\rho \left(W+\omega \cos\theta \right)\sin\left(Wt+\phi \right)\right] \end{array}\right\}$$

where $f_{0}$ is carrier frequency, c is speed of light, ρ is the radius of the conical base, d is the height of the center of gravity, *W* is the spin frequency, *ϕ* is the angular position of the scattering points at the conical bottom edge in the bottom disk, θ is the precession angle, ω is the precession angle frequency, α is the LOS angle, and $f_{D}$ is the micro-Doppler frequency.

Due to the superposition of echo signals from conical scattering points, the detailed information about the spectrum overlaps together, making it difficult to distinguish the corresponding frequency curves of different scattering points. However, the maximum value of the micro-Doppler frequency shift at each moment, namely the edge information about the spectrum, is the most obvious. Therefore, the maximum frequency shift is the key information for solving the micro-motion parameters. The expression for the maximum micro-Doppler frequency shift can be obtained by analyzing different time points in Equation (19) within one precession cycle.

When ωt=2mπ(m=0,1,2,…), the maximum frequency shift value is as follows:

(20) $$f_{DMax}=\frac{2f_{0}}{c}\rho \left|\omega \sin\alpha +W\sin\left(\alpha -\theta \right)\right|$$

When ωt=(2m+1)π(m=0,1,2,…), the maximum frequency shift value is as follows:

(21) $$f_{DMax}=\frac{2f_{0}}{c}\rho \left|\omega \sin\alpha +W\sin\left(\alpha +\theta \right)\right|$$

When ωt=(2m−1/2)π,(m=0,1,2,…), the maximum frequency shift value is as follows:

(22) $$\begin{array}{c} f_{DMax}=\frac{2f_{0}}{c}\left[\begin{array}{c} \rho W\cos\alpha \sin\theta \cos\Phi +d\omega \sin\alpha \sin\theta \\ \rho \left(W+\omega \cos\theta \right)\sin\alpha \sin\Phi \end{array}\right]\\ \Phi =\mathrm{Arctan}\frac{\left(W+\omega \cos\theta \right)\sin\alpha }{W\sin\theta \cos\alpha }+\pi \end{array}$$

When ωt=(2m+1/2)π,(m=0,1,2,…), the maximum frequency shift value is as follows:

(23) $$\begin{array}{c} f_{DMax}=\frac{2f_{0}}{c}\left[\begin{array}{c} \rho W\cos\alpha \sin\theta \cos\Phi +d\omega \sin\alpha \sin\theta \\ \rho \left(W+\omega \cos\theta \right)\sin\alpha \sin\Phi \end{array}\right]\\ \Phi =\mathrm{Arctan}\frac{\left(W+\omega \cos\theta \right)\sin\alpha }{W\sin\theta \cos\alpha } \end{array}$$

By reading the frequency values corresponding to four different time points within one precession cycle on the edge curve in the time–frequency spectrum, as shown in Figure 5, and then substituting the four frequency values as frequency shift maxima into Equations (20)–(23), four nonlinear equations with unknown cone parameters can be established.

### 3.2. Parameter Extraction and Analysis of Range Profile for the Micro-Motion Cone

According to the dynamics model of the micro-motion cone, the expression for the variation of projection distance of the cone vertex on the LOS with time is obtained as follows:

(24) $$p_{A}\left(t\right)=\left(h-d\right)\left(\cos\alpha \cos\theta +\sin\alpha \sin\theta \cos\omega t\right)$$

where h is the height of the cone and $p_{A}\left(t\right)$ is the radial projection distance of the cone top.

**Figure 6:**
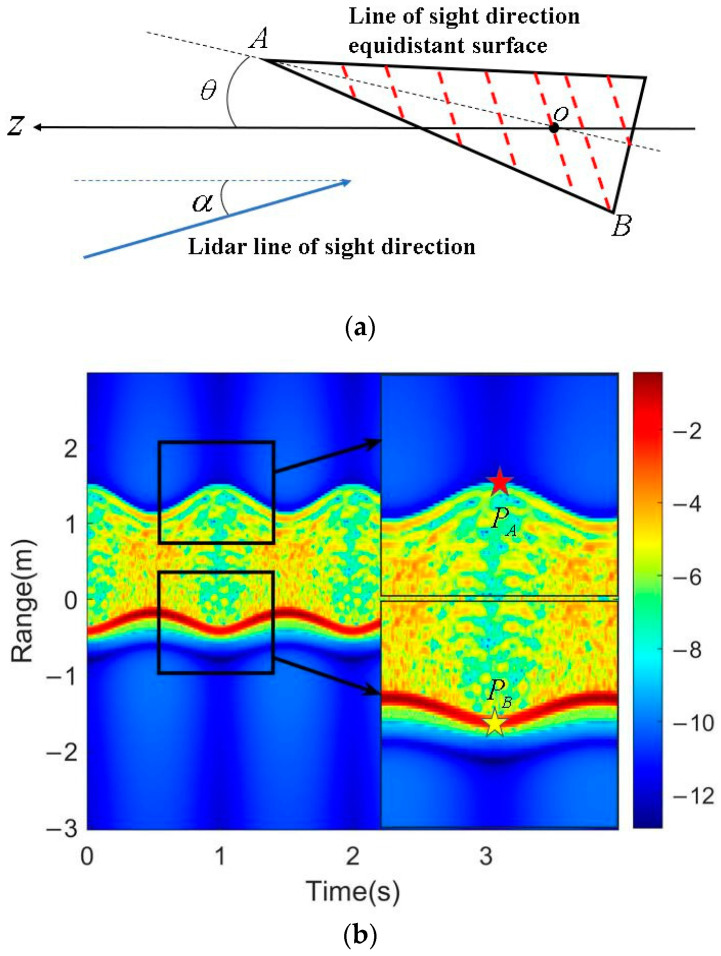
Schematic diagram and conical range–time spectrum. (**a**) Schematic diagram of conical projection distance; (**b**) schematic diagram of extracting feature points from conical range–time spectrum.

With the cone movements, the radial projection distance of the peak one-dimensional range profile also changes with time, corresponding to the equidistant surface B in Figure 6a. The expression of projection distance is as follows:

(25) $$p_{B}\left(t\right)=\sqrt{d^{2}+\rho^{2}}\cos\left[\arccos\left(\cos\alpha \cos\theta +\sin\alpha \sin\theta \cos\omega t\right)+\arctan\left(\frac{\rho }{d}\right)\right]$$

where $p_{B}\left(t\right)$ is the radial projection distance of the equidistant surface *B*.

When ωt=2mπ(m=0,1,2,…), the maximum values for $p_{A}\left(t\right)$ and $p_{B}\left(t\right)$ are described as follows, respectively:

(26) $$P_{A}(t)=(h-d)(\cos\alpha \cos\theta +\sin\alpha \sin\theta )$$

(27) $$P_{B}(t)=\sqrt{d^{2}+\rho^{2}}\cos\left[\arccos\left(\cos\alpha \cos\theta +\sin\alpha \sin\theta \right)+\arctan\left(\frac{\rho }{d}\right)\right]$$

Extraction results for the maximum values of the edge and peak curve from the range–time spectrum are shown in Figure 6b. By substituting the extracted distances into Equations (26) and (27), two nonlinear equations with unknown cone parameters can also be established.

Finally, the six nonlinear equations with unknown cone parameters established from Section 3.1 and Section 3.2 are combined and fitted by using the Levenberg–Marquardt optimization algorithm [30]. The initial solution and approximate range of solution is set; then, the optimal approximate solution is iteratively obtained by using the least squares method. By using the above method, the decoupling and extraction of micro-motion parameters and structure parameters can be achieved.

## 4. Simulation Verification of Conical Micro-Motion Parameter Extraction

The structure parameters of the precession cone, the micro-motion parameters, and the lidar parameters set in the simulation verification are shown in Table 2.

For the precession frequency extraction of the micro-motion cone, the empirical mode decomposition (EMD) method already exists for detection and estimation [6]. Therefore, the precession frequency *ω* and LOS angle α can be regarded as known quantities.

The estimated values of cone height, base radius, center of gravity height, spin frequency, and precession angle at different LOS and precession angles have been extracted. To evaluate the performance of the algorithm extraction, the *MAPE* factor (Mean Absolute Percentage Error) [31] is introduced to quantitatively describe the absolute deviation between the true and estimated values.

(28) $$MAPE=\frac{100\%}{N}\sum_{n=1}^{N}\left|\frac{y(n)-y}{y}\right|$$

where *N* is the number of Monte Carlo experiments, which is set to 100 here; *y* is the true value of the parameter, and *y*(*n*) is the estimated value of the parameter. The larger the *MAPE* value, the greater the error in parameter extraction. The smaller the *MAPE* value, the higher the accuracy of parameter extraction.

**Figure 7:**
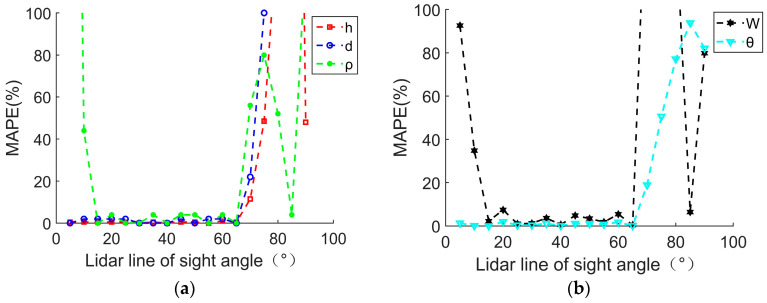
*MAPE* for extracting parameter values varies with LOS. (**a**) *MAPE* for extracting values of *h*, *d*, *ρ*; (**b**) *MAPE* for extracting values of *W*, *θ*.

The precession frequency is fixed to 2 Hz, and the LOS angle is set to increase from 5° to 90° with a step of 5°. The parameter extraction and *MAPE* analysis results are shown in Table 3 and Figure 7, where SNR = 15 dB. If the *MAPE* value is greater than 100, it is meaningless and will not be considered in Figure 7.

In actual detection, the lidar will not illuminate along the translational direction of the conical moving object, so the case of the LOS angle of 0° is not considered in the simulation. The results indicate that when the LOS angle is very small, the bottom radius *ρ* and the extraction value of the spin frequency *W* have a large error. When the LOS angle is large and close to 90°, the result of parameter extraction generates significant errors. When the LOS angle varies between 15° and 65°, the parameter extraction result is relatively accurate.

Figure 7 also clearly indicates that when the LOS angle approaches 0° and 90°, the obtained *MAPE* of the parameter extraction rapidly increases. In these cases, the parameter extraction method proposed in this paper is no longer applicable. The reason for this is that when the LOS angle is very small or close to 90°, the lidar transmission signal is incident downwards from the cone top (or perpendicular to the cone), and the minimum value of the conical edge curve for the distance–time spectrum no longer corresponds to the distance change at the cone top. Therefore, the parameter decoupling extraction method based on the distance edge curve is no longer accurate and has certain limitations. However, under the specific conditions (within a LOS angle ranging from 15° to 65°), the accuracy of parameter extraction is relatively high. The average absolute percentage errors of the five parameter extraction values are all found to be below 10%, meeting the requirements for further classification and recognition.

**Figure 8:**
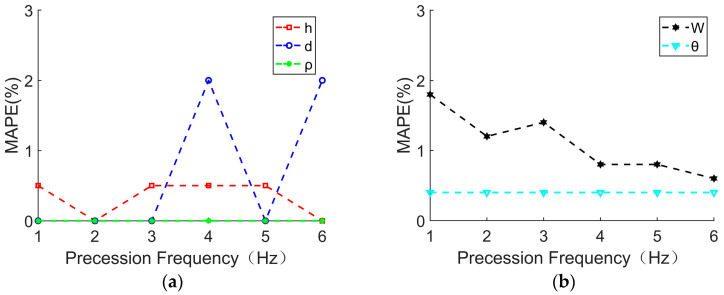
*MAPE* for extracting parameter values varies with precession frequency. (**a**) *MAPE* for extracting values of *h*, *d*, *ρ*; (**b**) *MAPE* for extracting values of *W*, *θ*.

The LOS angle is fixed to 30°, and the precession frequencies are increased from 1 Hz to 6 Hz with an interval of 1 Hz. The parameter extraction and *MAPE* analysis results are shown in Table 4 and Figure 8, where SNR = 15 dB. Similarly, if the *MAPE* obtained by the parameter extraction is greater than 100, it is meaningless and is not considered in Figure 8.

It can be seen from Figure 8 that within the applicable LOS angle range of the extraction algorithm, the parameters extraction error is almost not affected by the precession frequency changes. All five extracted parameters have no significant error deviation. The extraction results of the micro-motion parameters are relatively accurate. Therefore, the parameter decoupling extraction algorithm proposed in this paper is applicable within the LOS angle range of 15–65°, increasing the accuracy of the parameter’s extraction and maintaining an error of less than 10%.

As for this 10% error, possible influencing factors include the following: there is a reading error when reading the frequency value of the spectrum and the distance value of the distance profile due to the influence of the system signal-to-noise ratio (SNR); the approximation algorithm used to solve parameters through equations causes calculation errors; the resolution of the range profile and the resolution of the micro-Doppler time–frequency spectrum also have an impact on parameter extraction errors. In the future, parameters extraction errors can be further reduced by optimizing nonlinear equation approximation algorithms, improving the resolution of distance and velocity, or reducing the system signal-to-noise ratio.

## 5. Conclusions

In order to enrich the detection information about conical micro-motion objects, to reduce the extraction error of structure parameters, and to improve the classification and recognition accuracy of conical micro-motion objects, this paper proposed a new method for decoupling and extracting conical micro-motion parameters by combining the range profiles and micro-Doppler time spectrum. Firstly, the feasibility of obtaining range profiles of the micro-motion cone using FMCW lidar was verified by simulation. Based on the dynamic analysis of the micro-motion cone, the expression of the distance change between the cone top and the center of conical gravity, as well as the expression of the micro-Doppler frequency at the cone bottom, were given. Finally, the conical height, center of gravity height, base radius, spin frequency, and precession angle were extracted from simulation results under different LOS and precession angles. Furthermore, the errors between the extracted results and the true results were compared and analyzed. The results showed that the decoupling and parameter extraction methods had high extraction accuracy within the LOS range of 15°–65°, with an average absolute error of less than 10%. This method can effectively decouple and extract the structure and micro-motion parameters of cones, enriching the detection information and reducing parameter extraction errors and laying a theoretical and technical foundation for high-precision conical micro-motion classification and recognition.

## Figures and Tables

**Table 1:** Precession parameters and radar emission parameters.

Conical Precession Parameters		FMCW Lidar Transmission Parameters	
		Sweep bandwidth *B*	5 GHz
		Sweep period *T*	10 μs
Precession angle *θ*	15°	Transmit carrier frequency *f*_0_	2.82 × 10^14^ Hz
Lidar line of sight angle α	30°	Detection time *t*	4 s
Precession frequency ω	1 Hz	Carrier wavelength λ	1064 nm
Spin frequency *W*	3 Hz	Output power	300 mW
		CW radiation line width	10 kHz
		Output beam quality	1.2 M^2^
		Half beam divergence	0.019°

**Table 2:** Cone and lidar parameters.

	Parameters	Values
Cone	cone height *h*	2 m
	height of gravity center *d*	0.5 m
	bottom radius *ρ*	0.25 m
	precession angle θ	15°
	precession frequency ω	2 Hz
	spin frequency *W*	5 Hz
Lidar	sweep bandwidth *B*	5 GHz
	sweep period *T*	10 μs
	carrier frequency *f*_0_	2.82 × 10^14^ Hz
	detection time *t*	4 s

**Table 3:** Parameter extraction results for different LOS angles.

*α* (°)	*h* (m)	*d* (m)	*ρ* (m)	*W* (Hz)	*θ* (°)
5	2.01	0.50	1.58	0.37	14.81
10	1.99	0.49	0.36	3.26	15.02
15	2.01	0.51	0.25	5.11	15.01
20	2.01	0.51	0.24	5.37	14.72
25	1.99	0.49	0.25	4.95	15.06
30	2.00	0.50	0.25	4.94	15.06
35	1.99	0.50	0.26	4.82	15.17
40	2.00	0.50	0.25	4.97	15.02
45	1.99	0.51	0.24	5.24	14.83
50	1.99	0.50	0.26	4.83	15.15
55	2.00	0.49	0.25	4.91	15.12
60	1.98	0.49	0.26	4.73	15.25
65	1.99	0.50	0.25	5.02	14.98
70	2.23	0.61	0.11	13.72	12.17
75	2.97	1.00	0.05	30.48	7.41
80	4.87	2.16	0.12	11.99	3.44
85	12.89	7.95	0.24	5.32	0.93
90	2.96	2.75	0.57	1.01	2.69

**Table 4:** Parameter extraction results for different precession frequencies.

*ω* (Hz)	*h* (m)	*d* (m)	*ρ* (m)	*W* (Hz)	*θ* (°)
1	1.99	0.50	0.25	4.91	15.06
2	2.00	0.50	0.25	4.94	15.06
3	1.99	0.50	0.25	4.93	15.06
4	1.99	0.49	0.25	4.96	15.06
5	1.99	0.50	0.25	4.96	15.06
6	2.00	0.49	0.25	4.97	15.06

## Data Availability

Data are contained within the article.
